# Emergence and Phylogenetic Analysis of a Getah Virus Isolated in Southern China

**DOI:** 10.3389/fvets.2020.552517

**Published:** 2020-12-03

**Authors:** Tongwei Ren, Qingrong Mo, Yuxu Wang, Hao Wang, Zuorong Nong, Jinglong Wang, Chenxia Niu, Chang Liu, Ying Chen, Kang Ouyang, Weijian Huang, Zuzhang Wei

**Affiliations:** Laboratory of Animal Infectious Diseases and Molecular Immunology, College of Animal Science and Technology, Guangxi University, Nanning, China

**Keywords:** genetic analysis, phylogenetic analysis, isolation, emergence, Getah virus

## Abstract

Getah virus (GETV) has caused many outbreaks in animals in recent years. Monitoring of the virus and its related diseases is crucial to control the transmission of the virus. In the summer of 2018, we conducted routine tests on clinical samples from different pig farms in Guangxi province, South China, and isolated and characterized a GETV strain, named GX201808. Cytopathic effects were observed in BHK-21 cells inoculated with GX201808. The expression of E2 protein of GETV could be detected in virus-infected cells by indirect immunofluorescence assays. Electron microscopic analysis showed that the virus particles were spherical and ~70 nm in diameter with featured surface fibers. The multistep growth curves showed the virus propagated well in the BHK-21 cells. Molecular genetic analysis revealed that GX201808 belongs to Group 3, represented by Kochi-01-2005 isolated in Japan in 2005, and it clustered closely with the recently reported Chinese strains isolated from pigs, cattle, and foxes. A comparison of the identities of nucleotides and amino acids in the coding regions demonstrated that the GX201808 showed the highest amino acid identity (99.6%) with the HuN1 strain, a highly pathogenic isolate resulting in an outbreak of GETV infection in swine herds in Hunan province in 2017. In the present study, GETV was identified and isolated for the first time in Guangxi province of southern China, suggesting that future surveillance of this virus should be strengthened.

## Introduction

Getah virus (GETV) is an enveloped, single-stranded positive-sense RNA virus. GETV is a member of the genus *Alphavirus* in the family *Togaviridae*, which are transmitted mostly by various mosquito species (1). The virus comprises a genome of ~11.7 kb containing a 5′-untranslated region (UTR), two large open reading frames (ORFs), a 3′-UTR, and a poly-A tail (2). The ORF1 is situated at the 5′-end of the genome and encodes non-structural polyproteins (nsP1 to nsP4). The ORF2 is located at the 3′-end of the genome and encodes structural polyproteins that are transcribed into five structural proteins, namely, C, E3, E2, 6K, and E1, respectively (3, 4).

GETV has been shown to be distributed widely in the Asiatic, Australia, and Eurasian regions since the prototype GETV strain (MM2021) was first isolated from mosquitoes in Malaysia in 1955 (5–10). Sero-epizootiologic investigations showed that the virus is present in pigs, horses, goats, cattle, boars, and other animals including humans (9, 11–14), suggesting that the host range of the virus has expanded broadly. GETV infections can cause fever, rashes, and edema of the hindlegs in horses (15), fetal death and reproductive disorders in pigs (16) as well as fever, anorexia, depression, neurologic symptoms, and death in foxes (17). GETV infections in horses and pigs have been reported several times in Japan since the 1970's (18–21), and the outbreak of GETV infections in horses were reported in 1990 in India (22). In China, GETV is widely distributed in 15 provinces ranging from the southwest to northern areas of China since it was first identified from mosquitos in Hainan province, and it has caused several outbreaks in animals in recent years (2, 7, 17, 23–28). In 2017, an outbreak of GETV infection was reported in swine herds in Hunan province, China, resulting in the death of ~200 piglets and reproductive disorders of more than 150 pregnant sows (25). Lethal infections in blue foxes caused by GETV were also reported in Shandong province, East China (17). The latest GETV outbreak in racehorses occurred in Guangdong province, South China, in 2018 (27). Recently, serum samples from beef cattle showing sudden onset of fever have occurred in GETV-positive animals (26).

In this study, we conducted routine tests on clinical samples for GETV in samples from different farms in Guangxi province, South China, in 2018. A GETV strain was isolated from the serum of a GETV-positive animal. The virus was genetically closely related to recently isolated strains, HuN1, SD17/09, and JL1808 from different animals in Hunan, Shandong, and Jilin province, respectively, indicating a potential national emergence of this virus.

## Materials and Methods

### Cell Culture and Antibody Production

Baby hamster kidney cells (BHK-21; ATCC CCL10) were cultured in modified Eagle's medium (MEM) supplemented with 10% fetal bovine serum (FBS) as described in our previous study (29). To generate the E2 antibody against GETV, the E2 coding region was amplified by RT-PCR using forward primer (5′-CGGGATCCAGTGTGACGGAACACTT-3′) and reverse primer (5′-CCGGAATTCGGCATGCGCTCGTGGCGCGCA-3′). The forward and reverse primers carried the EcoR I and BamH I restriction sites, respectively. Thermal cycling conditions were 94°C for 3 min, followed by 30 cycles of 94°C for 30 s, 60°C for 30 s, 72°C for 90 s, and a final elongation step at 72°C for 10 min. The E2 PCR produced were double digested with the EcoR I and BamH I and then ligated into similarly digested pET-32a (+) expression vector (Novagen), resulting in plasmid pET32a-E2 and BL21 (DE3). *Escherichia coli* cells were transformed with pET32a-E2 and then induced by 1 mM IPTG for 4 h. A HIS binding kit (Novagen) was then used to purify the recombinant E2 protein. New Zealand white rabbits were injected with the purified recombinant E2 protein to generate a polyclonal antibody against the GETV E2 protein. Affinity chromatography with protein A was used to purify the polyclonal antibody (anti-GETV-E2 PcAb).

### Sample Collection, Viral RNA Extraction, and GETV Detection

Three hundred fifty field samples (sera) were collected from clinically diseased pigs in Guangxi province, South China. Viral RNA from 200 μl of each sample was extracted using the Prep Body Fluid Viral DNA/RNA Mini Prep kit (Axygen AXY) according to the manufacturer's instructions. The extracted RNA was then used for cDNA synthesis using M-MLV reverse transcriptase with random hexamers (Takara Bio, Inc., Dalian, China) according to the manufacturer's instructions. RT-PCR was then performed to detect GETV using primers as described in a previous study (23). Thermal cycling involved initially heating at 94°C for 3 min, followed by 35 cycles of 94°C for 30 s, 55°C for 30 s, and 72°C for 60 s, with a final extension step at 72°C for 10 min.

### Virus Isolation

The PBS-diluted GETV-positive sera were filtered through 0.22-μm filters (Millipore, Billerica, MA, USA) and plated onto BHK-21 cell monolayers seeded in a six-well plate. After 1 h of incubation at 37°C, the cells were washed twice with PBS and maintained in MEM supplemented with 2% FBS (Gibco) in a 5% CO_2_ incubator. The cells were observed on a daily basis for the presence of cytopathic effects (CPE). The supernatants (200 μl) were subsequently used for serial passages into BHK-21 cells. The third passage (P3) identified the presence of the virus by RT-PCR and indirect immunofluorescence assays (IFAs) using a polyclonal antibody against GETV E2 protein. The isolates were plaque purified three times and then used for complete genome sequencing.

### Plaque Assay

Viral plaque assays were performed using BHK-21 cells grown in six-well plates. Viral samples were serially 10-fold diluted in MEM. Samples, 200 μl, of each dilution were inoculated onto monolayers of BHK-21 cells and incubated for 1 h. The cells were then overlaid with a mixture of MEM containing 1% low-melting agarose (Cambrex, Rockland, ME, USA) and 2% FBS and incubated at 37°C for 3 days in 5% CO_2_. After careful removal of the medium, the cells were stained with 3–4 ml of staining solution consisting of 0.5% crystal violet and 25% formaldehyde solution for 15 min, and visible plaques were observed.

### Growth Curve

Viral growth kinetics were determined using BHK-21 cells as described previously (23). Briefly, BHK-21 cells in six-well plates were inoculated with GETV (P3) at a multiplicity of infection (MOI) of 0.1. After 1 h of incubation at 37°C, the BHK-21 cells were washed twice with PBS. Two hundred microliters of BHK-21 cell supernatants were harvested at 6, 12, 24, 36, 48, and 72 hpi and stored at −70°C until use. The virus titers (TCID_50_) for each time point were assessed using BHK-21 cells and calculated according to the Reed–Muench method. The growth curves were determined after measuring the mean titers of three independent measurements at each time point.

### Indirect Immunofluorescence Assay

The expression of viral proteins in GETV-infected BHK-21 cell was tested by IFAs. BHK-21 cell monolayers were inoculated with passaged viruses at one MOI. Twenty-four hours post-inoculation, the infected cells were washed twice with PBS, followed by fixation in cold acetone at −4°C for 30 min. The cells were washed five times with PBS and then blocked with 1% BSA (fraction V bovine serum albumin; Roche, Mannheim, Germany), which was diluted in PBS, for 30 min at room temperature. After being washed with PBS, the cells were incubated with primary anti-GETV-E2 PcAb (1:100) for 1 h at room temperature. Then the cells were washed with PBS five times followed by incubation with goat anti-rabbit IgG (H + L; Alexa (Fluor® 488, Abcam) for 1 h at 37°C. The cells were subsequently washed five times with PBS. Finally, images were captured using an inverted fluorescence microscope (Nikon, Tokyo, Japan).

### Preparation of Virus Particles and Electron Microscopy

BHK-21 cell monolayers were inoculated with the virus at one MOI. At 24 hpi, 30 ml of supernatant from the infected cells was harvested and filtered through 0.22-μm filters (Millipore, Billerica, MA, USA) and then mixed with 7.5 ml of 50% PEG-8000 to a final 10% concentration. The mixture was gently stirred at 4°C overnight and then centrifuged at 12,000 rpm at 4°C for 2 h. After careful removal of the supernatants, the precipitated viruses were re-suspended in 1 ml of TBS. The virus–TBS mixture was stirred at 4°C for 30 min for being negatively stained and was visualized by transmission electron microscopy (TEM).

### Complete Genome Determination

The viral genomic RNA was extracted from BHK-21 cells infected with GETV and then reversed transcribed into cDNA using M-MLV reverse transcriptase (TaKaRa, Dalian, China) according to the manufacturer's instructions. PCR was performed using TaKaRa LA Taq (TaKaRa, Dalian, China) to amplify the complete genomic sequence using the previously published PCR primers (23). The reaction conditions were 94°C for 3 min, followed by 30 cycles of 94°C for 30 s, 60°C for 30 s, 72°C for 90 s, and a final elongation step at 72°C for 10 min. The positive amplicons were purified using an E.Z.N.A.TM Gel Extraction kit (OMEGA, USA) and then inserted into a pMD18-T vector (TaKaRa, Dalian, China) for nucleotide sequencing in both directions using universal primers T7 and SP6. The genomic sequence of GETV was assembled using the SeqMan program of DNAstar software, version 7.0 (DNASTAR Inc., Madison, WI, USA).

### Sequence Alignments and Phylogenetic Analysis

Differences in sequence of this isolate and all other GETV strains available from GenBank were analyzed using the MegAlign program with DNAstar 7.0 software. The information regarding the reference GETV strains is listed in Table 1. Phylogenetic analyses were carried out based on the complete genome and E2 gene by MEGA version 6.0 using the maximum likelihood (ML) method with p-distances for nucleotide sequences, and the bootstrap test value was calculated using 1,000 replicates.

**Table 1:** Detailed information on the GETV strains in this study.

**Strain**	**GenBank number**	**Year**	**Host**	**Country**
MM2021	AF339484	1955	*C. gelidus*	Malaysia
Sagiyama virus	AB032553	1956	Mosquito	Japan
M1	EU015061	1956	*Culex* sp.	China
MI-110-C2	LC079087	1978	*Equus caballus*	Japan
MI-110-C1	LC079086	1978	*Equus caballus*	Japan
LEIV 17741 MPR	EF631999	2000	*Culex* sp.	Mongolia
LEIV 16275 Mag	EF631998	2000	Mongolia	Russia
HB0234	EU015062	2002	*Culex tritaeniorhynchus* Giles	China
South Korea	AY702913	2004	Swine	South Korea
YN0540	EU015063	2005	*Armigeres subalbatus*	China
Kochi/01/2005	AB859822	2005	*Sus scrofa*	Japan
HNJZ-S1	KY363862	2011	Pig	China
YN12031	KY434327	2012	*Armigeres subalbatus*	China
YN12042	KY450683	2012	*Culex tritaeniorhynchus* Giles	China
SC1210	LC107870	2012	*Armigeres subalbatus*	China
12IH26	LC152056	2012	*Culex tritaeniorhynchu*	Japan
14-I-605-C2	LC079089	2014	*Equus caballus*	Japan
14-I-605-C1	LC079088	2014	*Equus caballus*	Japan
HNJZ-S2	KY363863	2015	Pig	China
15-I-752	LC212972	2015	*Equus caballus*	Japan
15-I-1105	LC212973	2015	*Sus scrofa domesticus*	Japan
HNNY-1	MG865966	2016	Pig	China
HNNY-2	MG865967	2016	Pig	China
GETV-V1	KY399029	2016	Pig	China
16-I-676	LC223132	2016	*Equus caballus*	Japan
16-I-674	LC223131	2016	*Equus caballus*	Japan
16-I-599	LC223130	2016	*Equus caballus*	Japan
HNPDS-2	MG865969	2017	Pig	China
HNPDS-1	MG865968	2017	Pig	China
AH9192	MG865965	2017	Pig	China
JL17/08	MG869691	2017	Mosquito	China
JL1707	MH722255	2017	Mosquito	China
HuN1	MF741771	2017	Porcine	China
SD17/09	MH106780	2017	Fox	China
JL1808	MH722256	2018	Cattle	China
SC201807	MK693225	2018	Pig	China
GETV-GDFS2-2018	MT086508	2018	Pig	China

## Results

### Virus Detection, Isolation, and Plaque Purification

Routine tests were conducted on clinical samples collected from different pig farms in Guangxi province of southern China. Of the 350 field samples collected from the clinically diseased pigs in Guangxi province, South China, two serum samples from a swine herd were positive for GETV, as determined by specific RT-PCR (data not shown). One GETV-positive sample (GX201807) was collected from 42 weaning piglets of ~25 days old, which only exhibited fever for 1–2 days in a swine herd in Nanning, Guangxi Province, which has a total piglet population of 307. Another GETV-positive sample (GX201808) was collected from 12 pregnant sows suffering from reproductive disorders in a pig farm located in YuLin, Guangxi Province, which has a total sow population of 196. The GETV-positive sera samples were negative for PRRSV, SVV, PRV, JEV, and CSFV as demonstrated by RT/-PCR (data not shown).

**Figure 1:**
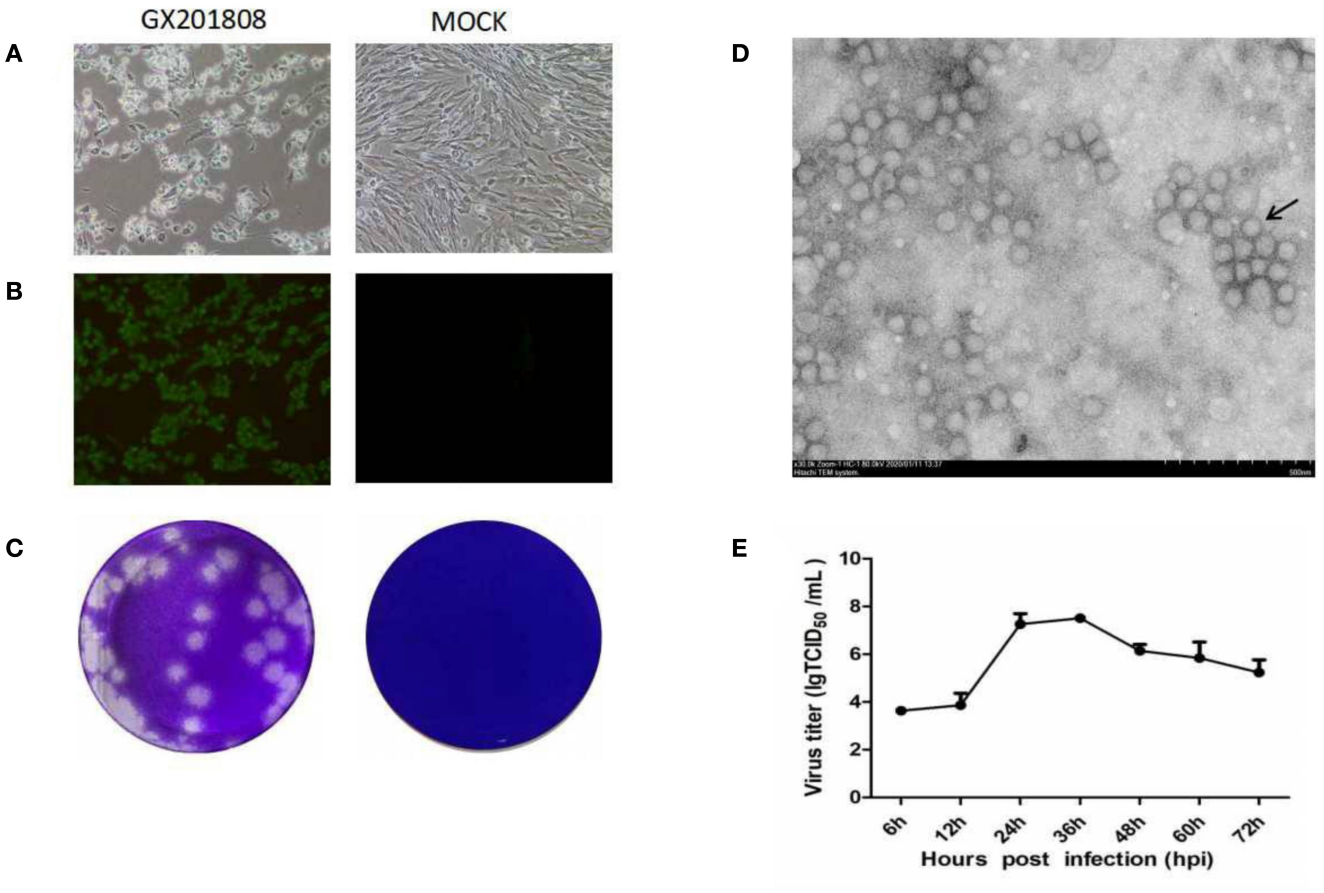
Isolation and identification of the Getah virus (GETV) GX201808 strain grown in baby hamster kidney (BHK-21) cells. **(A)** Cytopathic effects (CPEs) in BHK-21 cells infected with the GETV GX201808 strain. Mock-infected and virus-infected BHK-21 cells were observed at 36 hpi. **(B)** GETV GX201808 strain was identified in infected BHK-21 cells using immunofluorescence assay (IFA) at 24 hpi. The infected BHK-21 cells were fixed and stained using an anti-GETV-E2 PcAb against the GETV E2 protein and goat anti-rabbit H&L IgG. Images were taken using a 20x objective. **(C)** Plaque morphology of GETV GX201808 on BHK-21 cells. Monolayers of BHK-21 cells in six-well plates were infected with GETV GX201808. The cell monolayers were overlaid with 1% agarose and stained with crystal violet at 48 hpi. **(D)** Electron microscopic examination of morphology of GETV particles. BHK-21 cells were infected with GETV GX201808, and the precipitated viruses from the supernatants were processed for electron microscopy. **(E)** The growth of GETV GX201808 at a multiplicity of infection (MOI) of 1 on BHK-21 cells. The viral titers were determined as TCID_50_, and the values obtained were the means of three independent experiments.

Serum samples, which were GETV positive as determined by PCR, were inoculated into BHK-21 cells for virus isolation. CPE was generated in cells characterized by shrinkage, rounding, and detachment after 48 hpi (Figure 1A). The GETV isolates, named as GX201808, were obtained after serial passages and plaque purification in BHK-21 cells. The supernatants of each passage were GETV positive as confirmed by RT-PCR (data not shown). IFA analysis was conducted using an anti-GETV-E2 PcAb to confirm the isolation of the GETV strain. Figure 1B shows staining specific for E2, which was evident in GX201808-infected BHK-21 cells. The plaques generated by GETV in BHK-21 cells were regular in shape with distinct edges (Figure 1C). No viable particles were isolated in GX201807-inoculated BHK-21 cells, as confirmed by CPE, RT-PCR, and IFA (data no shown).

Electron microscopic examination of precipitated GX201808 strain particles revealed a cluster of typical morphology usually associated with alphaviruses. The virus particles were spherical with an average of 70 nm in diameter and featured surface fibers (Figure 1D). The multistep growth curves of the GETV strain was further analyzed using BHK21 cells. As shown in Figure 1E, the numbers of GX201808 particles exhibited a gradual increase from 6 hpi and reached a peak titer of ~10^8^TCID_50_ at 36 hpi. The titers then decreased slowly, reaching a titer of ~10^5^TCID_50_ at 72 hpi. These results revealed that a strain of GETV was successfully isolated when using BHK-21 cells.

### Genetic and Phylogenetic Analyses of the Virus

The full-length genome of the GETV strain GX201808 was sequenced and submitted to GenBank under accession no. MT269657. The entire genome of the GETV strain contained 11,691 bp in length excluding the polyA tail and possessed a typical alphavirus genome organization with 2 main ORFs, ORF1, and ORF2, and short UTRs at the 5′- and 3′-termini. All GETV strains available from GenBank were downloaded for sequence comparison and phylogenetic analysis. The results showed that GX201808 shared 94.9–99.4% sequence identity at the nucleotide level with other strains and the highest identity (99.4%) with strain SD17/09, which was recently isolated from foxes in China. The complete genome of GX201808 strain showed 97.3–99.3% identity with strains isolated from pigs in China, and only 97.6% identity with the strain (M1) first isolated in mosquitoes in China. Sequence comparison at the amino acid level of the GX201808 strain with other reported strains showed the sequence identities ranging from 97.4 to 99.7%, and 98.6 to 99.6% in the non-structural and structural polyproteins, respectively (Table 2).

**Table 2:** Nucleotide and amino acid sequences and identity analysis of GX201808 and the other GETV strains.

**Strains**	**GX201808 (%)**				
	**Complete genome**	**Non-structural polyprotein**		**Structural polyprotein**	
	**nt**	**nt**	**aa**	**nt**	**aa**
MM2021		94.6	97.4		
Sagiyama virus	96.9	96.5	98.0	97.1	99.1
M1	97.6	97.4	98.2	97.6	98.9
Sagiyama virus Original		96.6	98.3		
Ml-110-C1	98.1	98.2	99.2	98.0	99.5
MI-110-C2	98.1	98.2	99.3	98.0	99.4
LEIV16275 Mag	97.1	97.0	98.8	97.1	99.3
HB0234	97.5	97.4	98.9	97.4	98.9
Getah virus South Korea	97.8	97.9	99.2	97.7	99.4
Kochi-01-2005	99.0	99.0	99.4	97.4	99.2
YN0540	97.5	97.6	99.2	97.4	99.2
LEIV 17741 MPR	98.1	98.3	99.3	98.0	99.2
HNJZ-S1	97.4	97.5	99.3	97.3	99.0
121H26	97.4	97.5	99.2	97.2	99.1
SC1210	97.4	97.3	99.0	97.4	99.2
YN12031	95.9	95.9	98.0	95.9	98.6
YN12042	97.4	97.4	99.1	97.3	99.1
14-I-605-C1	97.3	97.4	99.2	97.2	99.1
14-I-605-C2	97.3	97.4	99.2	97.2	99.1
15-I-752	97.3	97.4	99.2	97.2	99.1
15-l-1105	97.3	97.4	99.1	97.2	99.0
HNJZ-S2	97.4	97.5	99.1	97.2	99.1
16-I-599	97.3	97.4	99.1	97.2	98.9
16-I-674	97.3	97.4	99.1	97.2	99.0
16-I-676	97.3	97.4	99.1	97.1	98.9
GETV-V1	97.4	97.4	99.0	97.4	99.1
HNNY-1	97.4	97.5	99.3	97.4	99.1
HNNY-2	97.4	97.5	99.3	97.3	99.1
AH9192	97.3	97.1	98.7	97.3	99.0
HNPDS-1	97.4	97.5	99.3	97.4	99.1
HNPDS-2	97.4	97.4	99.3	97.4	99.1
HuN1	99.3	99.4	99.6	99.2	99.4
JL1707	97.4	97.4	98.9	97.3	99.0
JL1708	97.4	97.4	99.1	97.3	99.1
SD17/09	99.4	99.3	99.6	99.4	99.5
JL1808	99.3	99.3	99.7	99.3	99.6
SC201807	97.3	97.4	99.2	97.2	99.2
GZ201808		97.1	98.7	97.2	99.1
GETV-GDFS2-2018	97.2	97.1	98.9	97.3	99.0

**Figure 2:**
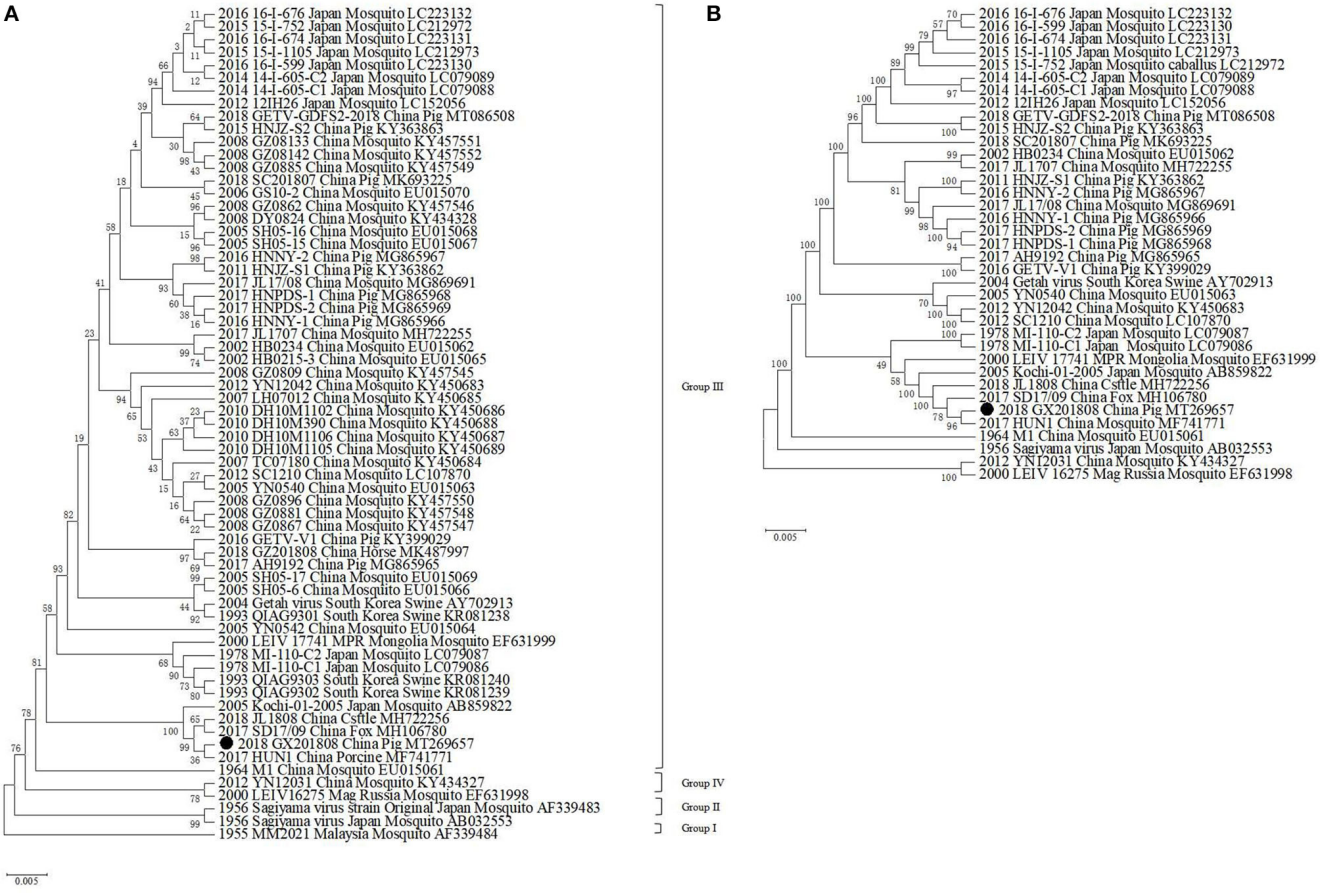
Phylogenetic analysis of GETV GX201808 with the available complete GETV genomes from the GenBank. The phylogenetic trees were generated using the neighbor-joining method implemented in the program MEGA 6.0. Bootstrap values are expressed as a percentage based on 1,000 replications. The strain obtained in this study is indicated by a closed triangle. **(A)** Diagrammatic representation of a tree based on the complete E2 gene nucleotide sequences of GETVs. **(B)** Diagrammatic representation of a tree based on the complete genome sequence of GETV GX201808 and the available complete GETV genomes from the GenBank.

Phylogenetic analysis showed that GETVs were divided into four evolutionary groups. Group I only had the oldest GETV strain (MM2021) isolated in 1963. Two GETVs strains isolated in Japan in 1956 formed Group II. Most of the GETV strains isolated from mosquitoes, pigs, horses, cattle, and other animals, including the strain GX201808 in this study were classified as Group III. Two GETV strains, YN12031 and LEIV/16275/Mag, were classified as Group IV (Figure 2).

## Discussion

GETV has a widespread geographic distribution, and the host range of the virus is expanding gradually (1, 3). Guangxi province of southern China is located in tropical and subtropical areas of the planet. The tropical and subtropical climates of these regions provide a favorable environment for the reproduction of mosquitoes, which play a key role in the spread of GETV among different hosts (30). The existence of GETV-infected animals and mosquitoes in Hunan, Guangdong, and Yunnan provinces, which are the neighboring provinces located in the east and west of Guangxi province, has meant that they are likely to spread, and they have already been reported (23, 25, 27, 28). In this study, we detected and isolated for the first time in Guangxi province, a GETV strain in pig sera using BHK-21 cells, proving the existence of GETV in this region of China. These GETV-positive samples were collected from weaning piglets exhibiting sudden onset fever or pregnant sows suffering from reproductive disorders.

Recently, an outbreak of GETV infection in swine herds resulted in reproductive disorders of pregnant sows and the death of piglets (25), indicating that this virus may pose a potential threat to swine health. Consistent with the findings of the previous study that reported the isolation of a strain of YN12031 (23), the GETV isolate GX201808 could generate CPE in BHK-21 cells characterized by shrinkage, rounding, and detachment. The virus could produce plaques and grow well in BHK-21 cells. Electron microscopic examination revealed that the virus particles were spherical with an average diameter of 70 nm, and they featured surface fibers, which was consistent with a previous study showing GETV particles display the typical morphology of alphaviruses (23, 31). The expression of E2 in the GX201808-infected cells could be detected by a specific polyclonal antibody raised against the E2 protein of GETV by IFA. This demonstrated that the GETV GX201808 strain was successfully isolated from BHK-21 cells. Viable GETV could not be isolated from BHK-21 cells inoculated with samples of GX201807, and this might be attributed to only a few virus particles in the samples or that the viruses had lost their viability during sample transportation from the pig farms to the laboratory.

It was shown that all the known GETV strains could be grouped into four evolutionary groups (24). The GX201808 strain was clustered in Group III and had the closest relationship with the HuN1 strain, which caused an outbreak of GETV infection in swine herds in Hunan province, China, leading to the death of piglets and reproductive disorders in pregnant pigs in 2017 (25). GX201808 also clustered closely with the recently reported Chinese GETV strains JL1808 and SD17/09 isolated from cattle and foxes, respectively (17, 26). Sequence comparison showed that GX201808 shares high sequence identity at the nucleotide level with the strains HuN1, SD17/09, and JL1808. Group III, represented by the first Chinese strain (M1) that was isolated in 1964 from mosquitoes, contains most GETV strains appearing in mosquitoes, pigs, horses, cattle, foxes, and other animals and has become the dominant viruses circulating among species (17, 23, 26–28).

An increase of high seroprevalence in pigs in the field was detected, and high virus titers were found in pigs infected experimentally with GETV, indicating that this species might be a natural reservoir and amplifiers of GETV (32–34). It has also been shown that GETV strains can circulate among pigs and horses simultaneously within the same region in Japan in 2015, and these were found to be closely related genetically (32). Recent studies also showed that mosquito-borne swine GETVs might play a role in transmitting GETV to blue foxes, cattle, and horses in different provinces of China (26, 27, 29), indicating that this virus has the potential to spread nationally and expand its host range. In response, we recommend that continuous surveillance of GETV infection in animals should be implemented in order to control the circulation of this potentially dangerous virus.

## Data Availability Statement

The datasets presented in this study can be found in online repositories. The names of the repository/repositories and accession number(s) can be found at: https://www.ncbi.nlm.nih.gov/genbank/, MT269657.

## Ethics Statement

The animal study was reviewed and approved by Ethics Committee of Guangxi University. Written informed consent was obtained from the owners for the participation of their animals in this study.

## Author Contributions

ZW conceptualized the study. TR and QM took part in the data curation. YW and ZN made the formal analysis. KO, YC, and WH were in charge of the investigation. HW, JW, and CN were in charge of the methodology. ZW was in charge of the project administration, wrote, reviewed, and edited the manuscript. All authors contributed to the article and approved the submitted version.

## Conflict of Interest

The authors declare that the research was conducted in the absence of any commercial or financial relationships that could be construed as a potential conflict of interest.
